# High pericardial and peri-aortic adipose tissue burden in pre-diabetic and diabetic subjects

**DOI:** 10.1186/1471-2261-13-98

**Published:** 2013-11-11

**Authors:** Fei-Shih Yang, Chun-Ho Yun, Tung-Hsin Wu, Ya-Ching Hsieh, Hiram G Bezerra, Chuan-Chuan Liu, Yih-Jer Wu, Jen-Yuan Kuo, Chung-Lieh Hung, Charles Jia-Yin Hou, Hung-I Yeh, Jason Jeun-Shenn Lee, Bernard E Bulwer, Ricardo C Cury

**Affiliations:** 1 Department of Radiology, Mackay Memorial Hospital, Taipei, Taiwan; 2 Department of Biomedical Imaging and Radiological Sciences, National Yang Ming University, Taipei, Taiwan; 3 Department of Anesthesia, Peking University First Hospital, Beijing, China; 4 Cardiovascular Department, University Hospitals Case Medical Center, Cleveland, USA; 5 Graduate Institute of Health Care Organization Administration, College of Public Health National Taiwan University, Taipei, Taiwan; 6 Health Evaluation Center, Mackay Memorial Hospital, Taipei, Taiwan; 7 Department of Medical Technology, Yuanpei University of Science and Technology, Hsin-Chu, Taiwan; 8 Department of Internal Medicine, Division of Cardiology, Mackay Memorial Hospital, Taipei, Taiwan; 9 Department of Medicine, Mackay Medical College, and Mackay Medicine Nursing and Management College, Taipei, Taiwan; 10 Institute of Traditional Medicine, National Yang-Ming University, Taipei, Taiwan; 11 Diagnostic Medical Sonography, Massachusetts College of Pharmacy and Health Sciences, Boston, Massachusetts, USA; 12 Noninvasive Cardiovascular Research, Cardiovascular Division, Brigham and Women’s Hospital, Boston, Massachusetts, USA; 13 Department of Radiology, Cardiovascular MRI and CT Program, Baptist Cardiac and Vascular Institute, Miami, FL, USA

**Keywords:** Pre-diabetes, Diabetes mellitus, MDCT, Pericardial adipose tissue, Peri-aortic adipose tissue, Insulin resistance

## Abstract

**Background:**

Central obesity in relation to insulin resistance is strongly linked to the development of type 2 diabetes. However, data regarding the association between pericardial and peri-aortic adiposity, a potential estimate of visceral adipose tissue burden, and pre-diabetes status remains unclear.

The aim of this study was to examine whether the degree of pericardial and thoracic peri-aortic adipose tissue, when quantified by multi-detector computed tomography (MDCT), differs significantly in a normal, pre-diabetic, and overtly diabetic population.

**Methods:**

We studied 562 consecutive subjects including 357 healthy, 155 pre-diabetic, and 50 diabetic patients selected from participants who underwent annual health surveys in Taiwan. Pre-diabetes status was defined by impaired fasting glucose or impaired glucose intolerance according to American Diabetes Association guidelines. Pericardial (PCF) and thoracic peri-aortic (TAT) adipose tissue burden was assessed using a non-contrast 16-slice multi-detector computed tomography (MDCT) dataset with off-line measurement (Aquarius 3D Workstation, TeraRecon, San Mateo, CA, USA). Body fat composition, serum high-sensitivity C-reactive protein (hs-CRP) level and insulin resistance (HOMA-IR) were also assessed.

**Results:**

Patients with diabetes and pre-diabetes had greater volume of PCF (89 ± 24.6, 85.3 ± 28.7 & 67.6 ± 26.7 ml, p < 0.001) as well as larger TAT (9.6 ± 3.1 ml vs 8.8 ± 4.2 & 6.6 ± 3.5 ml, respectively, p < 0.001) when compared to the normal group, although there were no significant differences in adiposity between the diabetic and pre-diabetic groups. For those without established diabetes in our study, increasing TAT burden, but not PCF, appear to correlate with insulin resistance (HOMA-IR) and hs-CRP in the multivariable models.

**Conclusions:**

Pre-diabetic and diabetic subjects, compared to normoglycemia, were associated with significantly higher pericardial and peri-aortic adipose tissue burden. In addition, visceral fat accumulation adjacent to the thoracic aorta seemed to exert a significant impact on insulin resistance and systemic inflammation.

## Background

Central obesity is a risk factor of metabolic syndrome, type 2 diabetes, and hyperlipidemia [1]. In the past decade, studies have focused on the relationship between metabolic derangements and regional fat deposits, particularly located in the trunk and waist area independent of total adiposity. Due to recent advances in radiological techniques, adiposity is readily assessable by computed tomography (CT), which may be a more direct measure of tissue burden. Based on this technique, more and more researches focus on ectopic visceral fat located between the myocardium and pericardium (pericardial) as well as those adjacent to the thoracic aorta (peri-aortic) in recent years. Visceral adipose tissues may play an important role in cardiovascular diseases and metabolic derangements such as diabetes, mainly due to the secretion of pro-inflammatory mediators and cytokines, as a consequence of the liver releasing of free fatty acids (FFAs) into the portal, leading to insulin resistance and systemic inflammation [2,3].

Pre-diabetes is characterized by impaired fasting glucose or impaired glucose tolerance status that reflects the stage of disordered glucose metabolism between normoglycemia and diabetes. Pre-diabetes is often under detected and remains asymptomatic, which may elevate future risks of diabetes and cardiovascular complications [4]. Recently, studies have shown that excessive adipose tissue deposits were closely related to diabetes development [5,6]. In addition, exaggerated systemic inflammation in response to excessive visceral adipose tissue had been proposed as the main mediating factor in pancreas functional failure, which plays a key role in type 2 diabetes. However, the relationship between visceral adipose tissue and pre-diabetes status before established clinical diabetes remains unknown. In this regard, the main objectives in this study were two-fold; first, we examined whether there are significant differences and distribution of PCF, TAT between subjects with pre-diabetes or diabetes. Second, we further aimed to examine whether VAT, either PCF or TAT, may still correlate several clinical cardiometabolic risks even in subjects without clinically overt diabetes.

## Methods

### Study subjects

The study was approved by the Institutional Review Board of Mackay Memorial Hospital, Taipei, Taiwan. The reference number is 09MMHIS028. All participants signed written informed consent prior to examinations. Data were analyzed anonymously. From 2005 to 2009, a total of 562 participants including 357 healthy, 155 with pre-diabetes and 50 with type 2 diabetes underwent health survey and received non-contrast enhanced computed tomography (CT) for assessment of cardiovascular risks by calculating coronary artery calcium in our center. All participants were consecutively enrolled using the following criteria and divided into three groups: normal (subjects without hypertension, type 2 diabetes, hyperlipidemia), pre-diabetes (impaired fasting glucose (IFG), impaired glucose intolerance, or IFG + IGT) and type 2 diabetes defined by the American Diabetes Association guidelines [7]. We further excluded subjects who had typical anginal symptoms during exercise or known cardiovascular diseases including myocardial infarction, coronary arterial disease, stroke, atrial fibrillation, prior hospitalization for congestive heart failure, and peripheral arterial disease.

### Demographic, anthropometric indices, and laboratory measures

Detailed physical examination was performed as well as a thorough review of baseline demographics, medical history including alcohol consumption, smoking, and physical activity status from structured questionnaires. All baseline characteristics and anthropometric measures including age, body height, body weight, waist, and buttock circumferences were all collected. Standardized sphygmomanometer cuff-defined resting blood pressures were measured under resting conditions by medical staff blinded to other test results. Body surface electrocardiogram (ECG) from 12-leads was performed for all subjects. The estimate of metabolic scores was calculated and presented as the numbers of abnormal items of the NCEP Panel III criteria (ATP III ) based on measures of waist circumference (Female > =80 cm or Male > =90 cm), fasting glucose (≥ 100 mg/dL), HDL cholesterol (Male < 40 mg/dL or Female < 50 mg/dL), triglyceride (> 150 mg/dL) and blood pressure (> 130/85 mmHg). High-sensitivity C-reactive protein (hs-CRP) levels were determined by using a highly sensitive, latex particle-enhanced immunoassay Elecsys 2010 (Roche, Mannheim, Germany).

### Glucose metabolism

All sample collection and analytic principles were based on the standard requirements according to the Clinical Laboratory Standards Institute (CLSI) guidelines (Specimen Choice, Collection, and Handling; Approved Guideline H18-A3). To ensure accuracy, samples had repeated tests in their original tubes within one day to avoid sample mix up. Normoglycemia was defined as fasting serum glucose (FSG) less than 100 mg/dL. Pre-diabetes was defined as impaired fasting glucose (IFG) and/or impaired glucose tolerance (IGT) using the American Diabetes Association diagnostic criteria [7]. Homeostasis model assessment of insulin resistance (HOMA-IR) was calculated.

### CT scan and quantification of pericardial and thoracic peri-aortic fat

MDCT of the coronary calcium was performed using a 16-slice MDCT scanner (Sensation 16, Siemens Medical Solutions, Forchheim, Germany) with 16 x 0.75 mm collimation, rotation time 420 ms and tube voltage of 120 kV. In one breath-hold, images were acquired from above the level of the tracheal bifurcation to below the base of heart using prospectively ECG triggering with the centre of the acquisition at 70% of the R-R interval. Visceral adipose tissue, PCF and TAT, was quantified by MDCT using a dedicated workstation (Aquarius 3D Workstation, TeraRecon, San Mateo, CA, USA). The semi-automatic segmentation technique was developed for quantification of fat volumes. We traced the region of interest manually and defined fat tissue as pixels within a window from -195 HU to -45 HU and a window center at -120 HU. PCF was defined as any adipose tissue located within the pericardial sac. TAT tissue was defined as all of the adipose tissue surrounding the thoracic aorta, which extended 67.5 mm from the level of the bifurcation of pulmonary arteries with cranial-caudal coverage of the thoracic aorta. This approach has previously been validated [6,8,9]. Initially, the intra-observer and inter-observer coefficient of variation were 4.27%, 4.87% and 6.58%, 6.81% for PCF and TAT, respectively. Both observers performed an independent reading in a random subset of 40 subjects [6].

### Statistical analysis

Continuous data were expressed as the mean and standard deviation with categorical data expressed as the frequency and proportion of occurrence. Differences in baseline demographics between groups were tested by Student t- test with categorical data analyzed by chi-square or Fisher’s exact test as appropriate. Wilcoxon non-parametric trend test was used to estimate the trend of all continuous data and ordinal variables across all ordered groups. Univariable logistic regression model was used to determine the significant factors in the prediction of different metabolic components stratified by both visceral adipose tissue and hs-CRP tertiles with individual odds ratio (OR), significance (p value), and 95% confidence interval (CI) reported. A multivariable regression model in subjects without overt diabetes was conducted to identify the independent role of visceral adipose in predicting hs-CRP level and HOMA-IR after adjustment for baseline clinical variables, various body size estimates, and biochemical data. The variables enrolled in multivariable models were chosen from those clinical covariates with significant associations with both visceral adipose tissue in univariate regressions. Due to collinearity, various body size estimates including BMI, BSA, waist circumference and body fat composition information were seqeuntially entered into multivariable models. All data was analyzed by commercialized software STATA 8.2 package (STATA Corp., College Station, Texas). The significance of p level (α-value) for all analysis was two-sided with 0.05 considered to be statistically significant.

## Result

### Study sample characteristics

**Figure 1:**
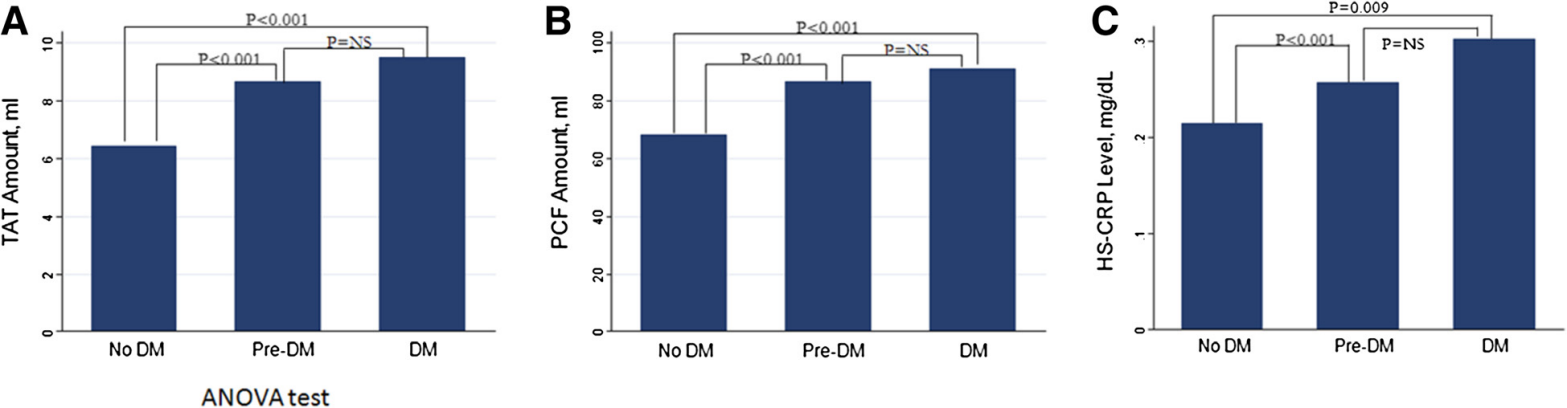
The correlation between normal, pre-diabetic, diabetic subjects and TAT (A), PCF (B) and hs-CRP (C).

Table 1 describes the demographic anthropometric and laboratory measures for all subjects included in this study. A total of 562 participants were enrolled: 357 healthy, 155 with pre-diabetes and 50 with type 2 diabetes. Both pre-diabetic and type 2 diabetic subjects were significantly older with higher blood pressure, weight, body mass index, waist circumferences, waist-to-hip ratio than the normal group (all p < 0.001). While no significant differences in body fat composition were found between subjects with pre-diabetes and normal subjects. In addition, triglyceride, fasting glucose, postprandial glucose, HbA1c, and HOMA-IR were higher with a lower HDL cholesterol level in subjects with pre-diabetes and type 2 diabetes, when compared with normal ones (all p < 0.001). Patients who had type 2 diabetes had differed significantly from ones with pre-diabetes with higher triglyceride, fasting glucose, postprandial glucose, HbA1c, and HOMA-IR as well as had lower HDL cholesterol levels. The mean value of PCF and TAT were 85.3 ± 28.7 & 8.8 ± 4.2 ml in pre-diabetic subjects and 89 ± 24.6 & 9.6 ± 3.1 ml in type 2 diabetic subjects, respectively, and were significantly higher than both VAT in normals (67.6 ± 26.7 ml & 6.6 ± 3.5 ml, all p < 0.001). However, both VAT were not different between pre-diabetic and type 2 diabetic subjects in Figure 1A and B. The difference among normal, pre-diabetic, and type 2 diabetic subjects for hs-CRP are shown in Figure 1C. Subjects with pre-diabetes and type 2 diabetes had higher hs-CRP than normals (all p < 0.001). There was no significant difference between pre-diabetes and type 2 diabetes in hs-CRP.

**Table 1:** The baseline demographic data of participants according to glucose tolerance status

	**No diabetes**	**Pre-DM**	**DM**	
	**N = 357**	**N = 155**	**N = 50**	**Trend P**
Age	47.5 ± 7.7	51 ± 8^*^	53.5 ± 8^*^	<0.001
Gender, female	127 (35.6%)	22 (14%)	12 (24%)	<0.001
SBP, mmHg	116.9 ± 15.4	124.6 ± 16.8^*^	130.2 ± 16.7^*^	<0.001
BMI, kg/m^2^	23.6 ± 3.1	25.3 ± 3.1^*^	26.2 ± 3.8^*^	<0.001
BSA, m^2^	1.72 ± 0.17	1.8 ± 0.16	1.81 ± 0.15	<0.001
WC, cm	81 ± 8.9	86.9 ± 8.3^*^	89.2 ± 8.7^*^	<0.001
Body Fat, %	24.9 ± 6.2	25.9 ± 6.2	27.3 ± 9.1^*^	0.08
PCF, ml	68.2 ± 25.5	86.8 ± 26.9	91 ± 24.1	0.005
TAT, ml	6.4 ± 3.3	8.7 ± 4.2	9.5 ± 3.2	0.005
Hypertension	30 (7.7%)	27 (17.5%)	17 (34%)	<0.001
Hyperlipidemia	14 (3.6%)	9 (5.8%)	5 (10%)	0.157
Smoking	83 (21.3%)	38 (24.7%)	16 (32%)	0.172
Fasting Glucose, mg/dL	91.6 ± 5.7	103.6 ± 8.6^*^	159.7 ± 52.7^*¥^	<0.001
Post-prandial Glucose, mg/dL	99.3 ± 15.7	117.5 ± 30.1^*^	205.2 ± 85.9^*¥^	<0.001
HbA1c, %	5.67 ± 0.32	5.93 ± 0.41^*^	7.52 ± 1.85^*¥^	<0.001
HOMA-IR	1.25 ± 0.85	1.81 ± 1.09^*^	3.42 ± 2.2^*¥^	<0.001
Uric Acid, mg/dL	5.58 ± 1.37	6.35 ± 1.48^*^	6.04 ± 1.28Υ	<0.001
Total Cholesterol, mg/dL	192.5 ± 33.9	195.1 ± 31.9	191.2 ± 39.7	0.79
TG, mg/dL	126.4 ± 65.1	145.3 ± 85.6^*^	204.6 ± 158.5^*¥^	<0.001
LDL cholesterol, mg/dL	125.2 ± 31	128.8 ± 31	122.8 ± 32	0.67
HDL cholesterol, mg/dL	53.5 ± 13.5	49.8 ± 13.4^*^	44.1 ± 9.3^*¥^	<0.001
BUN, mg/dL	11.9 ± 3.6	12.6 ± 3.4	13.1 ± 3.7^Υ^	<0.001
eGFR, mL/min/1.73 m^2^	86.9 ± 14.4	81.5 ± 13.3^*^	84.8 ± 20.2	0.31

ANOVA: ^*^p < 0.05 compared to No Diabetes group, ^¥^p < 0.05 compared to Pre-DM group, ^Υ^p > =0.05 & <0.1 compared to No Diabetes group. *BMI* body mass index, *HbA1c* glycosylated hemoglobin, *TG* triglyceride, *LDL cholesterol* low-density cholesterol, *HDL cholesterol* high-density cholesterol, *eGFR* estimated glomerular filtration rate, *WC* waist circumference.

In Table 2, we examined the association between both VAT and clinical continuous and dichotomous variables for subjects without established type 2 diabetes (N = 512). We observed that both VAT were positively associated with increasing age, and body size estimates (including BMI, BSA, waist circumference), body fat composition, systolic blood pressure, and fasting glucose level (all p < 0.001). Furthermore, both VAT were associated with higher triglyceride, lower HDL cholesterol (both p < 0.001) and higher LDL cholesterol (both p = 0.004). In Table 3, we further examined these associations in multivariable models. Both VAT were associated with higher HOMA-IR, hs-CRP in univariable model. When age, gender, various anthropometric measures, and clinical variables were further adjusted, we observed that the relationship between PCF and HOMA-IR, hs-CRP remained borderline (p > =0.03& <0.1). Increasing TAT burden correlated with higher HOMA-IR, hs-CRP (all p < 0.05) in the multivariable models.

**Table 2:** The regression models for visceral adipose tissue with clinical continuous and dichotomous risk variables for subjects without overt diabetes (N = 512)

	**PCF (ml)**		**TAT (ml)**	
	**β-Coef.**	**p value**	**β-Coef.**	**p value**
Age, years	0.34	<0.001	0.34	<0.001
Gender, female	−0.19	<0.001	−0.38	<0.001
SBP, mmHg	0.3	<0.001	0.35	<0.001
BMI, kg/m^2^	0.55	<0.001	0.57	<0.001
BSA, m2	0.45	<0.001	0.51	<0.001
WC, cm	0.58	<0.001	0.64	<0.001
Body Fat, %	0.3	<0.001	0.2	<0.001
SBP, mmHg	0.3	<0.001	0.35	<0.001
Fasting glucose, mg/dL	0.22	<0.001	0.24	<0.001
Total cholesterol, mg/Dl	0.08	0.05	0.05	0.216
HDL cholesterol, mg/dL	−0.24	<0.001	−0.36	<0.001
Triglyceride, mg/dL	0.18	<0.001	0.26	<0.001
LDL cholesterol, mg/dL	0.12	0.004	0.12	0.004
eGFR, mL/min/1.73 m2	−0.12	0.04	−0.22	<0.001
Hypertension	0.18	<0.001	0.22	<0.001
Hyperlipidemia	0.09	0.015	0.08	0.027
Exercise	−0.08	0.019	−0.11	0.002
Alcohol use	0.004	0.935	0.1	0.015
Smoking	0.06	0.082	0.12	0.001

*BMI* body mass index, *HbA1c* glycosylated hemoglobin, *TG* triglyceride, *LDL cholesterol* low-density cholesterol, *HDL cholesterol* high-density cholesterol, *eGFR* estimated glomerular filtration rate, *WC* waist circumference.

**Table 3:** The association of visceral adipose tissue with HOMA-IR, and Hs-CRP in subjects without overt diabetes (N = 512)

	**PCF (ml)**		**TAT (ml)**	
	**HOMA_IR**	**hs-CRP**	**HOMA_IR**	**hs-CRP**
	**β-Coef. (p value)**	**β-Coef. (p value)**	**β-Coef. (p value)**	**β-Coef. (p value)**
Un-adjusted Model	0.21 (<0.001)	0.16 (0.005)	0.43 (<0.001)	0.27 (<0.001)
Adjusted for BMI				
Age, gender, BMI	0.11 (0.089)	0.12 (0.582)	0.42 (<0.001)	0.17 (0.021)
Age, gender, BMI, Clinical variables	0.08 (0.266)	0.14 (0.373)	0.36 (0.005)	0.22 (0.024)
Adjusted for BSA				
Age, gender, BSA	0.1 (0.056)	0.18 (0.057)	0.43 (<0.001)	0.19 (0.006)
Age, gender, BSA, Clinical variables	0.05 (0.472)	0.08 (0.381)	0.36 (<0.001)	0.20 (0.026)
Adjusted for WC				
Age, gender, WC	0.06 (0.387)	0.11 (0.034)	0.41 (<0.001)	0.12 (0.002)
Age, gender, WC, Clinical variables	0.05 (0.514)	0.06 (0.482)	0.38 (<0.001)	0.20 (0.038)
Adjusted for body fat %				
Age, gender, body fat %	0.14 (0.049)	0.13 (0.659)	0.46 (<0.001)	0.22 (0.002)
Age, gender, body fat %, Clinical variables	0.09 (0.209)	0.13 (0.138)	0.40 (<0.001)	0.25 (0.007)

*BMI* body mass index, *HbA1c* glycosylated hemoglobin, *TG* triglyceride, *LDL cholesterol* low-density cholesterol, *HDL cholesterol* high-density cholesterol, *eGFR* estimated glomerular filtration rate, *WC* waist circumference.

Clinical variables included age, sex, systolic blood pressure, fasting glucose, triglyceride, *HDL cholesterol*, exercise, alcohol use, smoking, and hypertension or hyperlipidemia treatment.

## Discussion

Our study provides new insights into the understanding of the association of visceral adipose tissue, PCF and TAT, among persons with pre-diabetes, type 2 diabetes, and normoglycemia. It has been demonstrated that people with pre-diabetes are at significantly more risk of developing cardiovascular disease than those with normoglycemia, and will likely develop type 2 diabetes without intervention [10]. Central or visceral obesity is associated with increased insulin resistance, type 2 diabetes, hypertension, and hyperlipidemia. In an earlier work, Stancakova et al. [11] found that an increased waist circumference, the estimation of visceral adiposity in male among pre-diabetes. Recently, the association between diabetes and pericardial/epicardial adiposity measured by echocardiography and MDCT has been examined in several studies [2,6,12]. Using echocardiography, Iacobellis et al. observed a significant association between epicardial fat thickness and fasting blood glucose [12]. However, most of these studies did not show a significant difference in visceral adiposity in individuals with pre-diabetes compared to those with type 2 diabetes. Our data adds value to previous reports particularly establishing the association between visceral adiposity and glucose intolerance. Compared to a previous study [12], we used volume-based measures for assessing region-specific visceral adipose tissue surrounding the heart and thoracic aorta by multi-detector computed tomography, a more precise measure. Additionally, we extended these previous observations by showing that PCF and TAT are significantly increased in pre-diabetic individuals compared to normals, with no significant differences observed between individuals with type 2 diabetes and those with pre-diabetes. Interestingly, we observed similar trends in the associations regarding both region-specific VAT and several clinical metabolic risks and clinical co-variates as previous report in our current cohort [13]. This information suggests progressive metabolic derangements with increasing degrees of visceral adiposity, leading to a progression from early glycemic dysfunction to the pre-diabetes stage, in tandem with increasing CVD risk. The volume-based, three-dimensional CT measurement may be a useful tool for diabetes-related cardiovascular risk stratification in selected subjects.

How is visceral fat related to glucose deregulation? Increased abdominal visceral adiposity, rather than peripheral subcutaneous adiposity, has been associated with glucose intolerance or frank diabetes [14]. Bays et al. [15] has hypothesized the pathologic role of visceral adipose tissue as “sick fat”. This hypothesis states that “adiposopathy” is promoted by positive caloric balance and sedentary lifestyle in genetically susceptible individuals. The accumulation of visceral adipose tissue is associated with adverse endocrine and immune consequences due to released substances such as free fatty acids, leptin, adiponectin, pro-inflammatory agents, and decreased anti-inflammatory factors. As a result, it often results in unfavorable glucose metabolism and type-2 diabetes [15,16]. Also suggested is the possible role of lipids in beta-cell deterioration that leads to glucose intolerance [17]. Evidence from previous studies [18,19] has demonstrated that adverse metabolic derangements of excess fat are more closely related to the location than to the amount. Visceral adipose tissue and pericardial fat exhibit differences in leptin, adiponectin, and IL-6 secretion. However, a comprehensive characterization of pericardial and peri-aortic adipose tissue have not been established [20].

In our study, we also found that an interaction between regional-specific visceral adipose tissue and systemic inflammation in subjects without established diabetes, with only TAT but not PCF having a pronounced effect on HOMA-IR and hs-CRP through multivariate regression analysis. These results suggest that perivascular fat deposition can be implicated in systemic inflammation more strongly than pericardial fat deposition. Several possible mechanisms could be put forward in regard to the differential behavior of region-specific visceral adipose tissue.

First, PCF has been confined to the pericardial sac, but TAT surrounds the aorta, which is more prone to systemic effects via adventitial inflammation that traverse the arterial wall [21] and systemic inflammation impacting HOMA-IR and hs-CRP. Second, relatively healthy subjects presenting for health checkups without definite type 2 diabetes in our subanalyses may explain the lack of significant correlation of hs-CRP, HOMA-IR and PCF in multivariate analyses, which differ from those obtained in studies on diseased subjects [9,22].

Our study suggests that PCF and TAT have strong associations with glucose intolerance and type 2-diabetes. However, this cross-sectional study cannot assess clinical outcomes with respect to the development of type 2 DM since a large pre-diabetic population and long term follow up is needed for adequate statistical analysis. Future prospective trials are required to assess the prognostic value of CT-measured region-specific VAT for comparison with traditional risk factors.

There are several limitations of our study. Firstly, we included fewer women than men (male/female: 401/161), which may limit its generalizability and hardly performing the subsequent analyses for men and women separately, In addition, as in every cross-sectional study, we cannot pride the follow up data related to the relationship between region-specific VAT and progression of glycemic dysfunction. Future longitudinal studies of visceral adipose tissue burden in this population may help to clarify these relationships.

## Conclusions

Our data indicated that pre-diabetic status was associated with significantly higher pericardial and peri-aortic adipose tissues than normal subjects, which is actually comparable to established diabetic patients. In addition, in subjects without established diabetes, visceral fat adjacent to the aorta seemed to exert effects on insulin resistance and systemic inflammation. We believe that the major implications of this study are as follows: (1) increased specific regional visceral fat deposits may be related to pre-diabetes and could be used as additional information for cardiovascular risk stratification during the early stages of glucose dysfunction; (2) peri-aortic fat may exert a more significant systemic effect than pericardial adipose tissue.

## Competing interests

The authors declare that they have no competing interests.

## Authors’ contribution

FY, CY, CH and BB have substantial contributions to conception and design. CH, TW, YH, CL, JK, YW have participated in acquisition of data, or analysis and interpretation of data. HB, CJH, HY, JJL, RC have been involved in drafting the manuscript and revising it. All authors read and approved the final manuscript.

## Pre-publication history

The pre-publication history for this paper can be accessed here:

http://www.biomedcentral.com/1471-2261/13/98/prepub
